# The frequency and complexity of pediatric hospitalizations to pediatric and adult departments in Germany

**DOI:** 10.1186/s12887-026-06816-4

**Published:** 2026-04-08

**Authors:** Nora Bruns, Christian Dohna-Schwake, Christoph Härtel, Burkhard Rodeck, Simone Schwarz, Ursula Felderhoff-Müser, Rayan Hojeij

**Affiliations:** 1 Department of Pediatrics I, Neonatology, Pediatric Intensive Care Medicine, Pediatric Neurology, and Pediatric Infectious Diseases, University Hospital Essen, University of Duisburg-Essen, Hufelandstr. 55, Essen, 45239 Germany; 2 Centre for Translational Neuro-and Behavioural Sciences, University Hospital Essen, C-TNBS, University of Duisburg-Essen, Essen, Germany; 3 Department of Pediatrics, University Hospital Würzburg, University of Würzburg, Würzburg, Germany; 4 Christliches Kinderhospital Osnabrück, Osnabrück, Germany

**Keywords:** Ppediatric hospital care, Pediatric complex chronic conditions system, Pediatric departments, Adult departments

## Abstract

**Background:**

Germany’s upcoming hospital reform aims to restructure inpatient services by assigning hospitals to defined “care groups” that determine which services they may provide in the future. While pediatric care will continue to receive infrastructural readiness funding, the reform eliminates a dedicated care group for specialized pediatric medicine. As a result, subspecialized pediatric services will no longer be distinctly designated or planned for. This has raised concerns about future access to specialized inpatient care for children and adolescents, given the complexity and diversity of pediatric conditions. The aim of this study was to contribute objective data to the ongoing debate by investigating how pediatric patients are distributed between pediatric (PD) and adult departments (AD) and assessing case complexity.

**Methods:**

We conducted a retrospective observational study using data from the German nationwide hospital discharge dataset from 2016 to 2022, comprising all public hospitals. Hospitalized cases aged >28 days and <18 years were included and classified as surgical or non-surgical based on operation and procedure codes, while department classification relied on department codes. The main outcome was admission to pediatric versus adult department. Case complexity was assessed using the Pediatric Complex Chronic Conditions (PCCC) score and an exploratory modified PCCC model incorporating a logistic S-curve to reflect increasing complexity with multiple chronic conditions.

**Results:**

Of 7,896,283 analyzed pediatric cases, 74.1% were treated in PDs, 23.4% in ADs, and 2.5% in interdisciplinary settings. While 87.0% of non-surgical cases were treated in PDs, 58.9% of surgical cases were managed in ADs. Case complexity was highest in interdisciplinary cases, followed by PDs and ADs. The modified PCCC revealed a deflation of cumulative complexity in ADs and inflation in PDs, suggesting an underestimation of complexity from chronic conditions in standard scoring. PDs consistently treated more complex non-surgical and surgical cases.

**Conclusions:**

The majority of pediatric patients—especially those with complex non-surgical conditions—were treated in pediatric departments. These findings highlight the central role of pediatric inpatient structures in managing complex care needs. Preserving the visibility and planning relevance of pediatric subspecialty care is essential to safeguard age-appropriate, complexity-appropriate services for children and adolescents.

**Supplementary Information:**

The online version contains supplementary material available at 10.1186/s12887-026-06816-4.

## Introduction

The German hospital reform, announced by the federal government in 2023, aims to centralize specialist medical care by restructuring inpatient services into so-called “care groups”, which define the services hospitals may provide. These care groups will, as in the current system, be linked to both infrastructural readiness payments and case-based reimbursement. The planned catalog comprises 61 care groups, including general pediatrics (> 28 days to < 18 years), general pediatric surgery, hemato-oncology, and perinatology [1, 2].

A key structural change of the reform lies in the elimination of a dedicated care group for specialized pediatric medicine. As a consequence, hospitals with established pediatric subspecialty expertise beyond general pediatrics, neonatology, and hemato-oncology would no longer be distinctly designated or planned for within the new framework. Yet, pediatric subspecialization has historically been essential for providing appropriate structures, expertise, and multidisciplinary coordination for children with complex, rare, or organ-specific conditions. This change has therefore sparked substantial controversy among pediatric stakeholders, who express concern that highly specialized pediatric care may be de-emphasized within the new system and that more children could consequently be treated in adult departments.

To understand these concerns, it is important to acknowledge that pediatric subspecialties are as diverse as those in adult medicine and that the breadth of pediatric conditions is similarly wide; indeed, more than 500 diagnoses are listed in the International Classification of Diseases (ICD)-10. Moreover, pediatric medicine must account for age-specific anatomical and physiological differences, neurodevelopmental aspects, and the unique dynamics of the patient–parent dyad, all of which require dedicated expertise and often specialized—and costly—equipment. The rising number of young adults surviving childhood-onset conditions and transitioning to adult care adds further challenges to healthcare systems that may lack the structural and professional preparedness to meet their needs [3, 4].

Against this background, a more rational and evidence-based discussion about the role of specialized pediatric care in the context of the German hospital reform requires objective data on where children are actually treated and how complex these cases are. This study therefore aimed to assess the distribution of pediatric cases between pediatric (PD) and adult departments (AD) and to quantify the complexity of treated conditions using the nationwide German hospital discharge dataset, differentiating between surgical and non-surgical cases.

## Methods

This is a retrospective observational study using nationwide routine health care data from the German hospital dataset (GHD). Cases were identified via age at admission and analyzed with respect to the treating department (pediatric versus adult) and performed surgery (non-surgical versus surgical). The study included the datasets from 2016 to 2022 [5–11].

### Data source

The GHD is a nationwide dataset that contains all discharges from public hospitals in Germany. As there are no private children’s hospitals in Germany, the dataset represents a full survey of pediatric hospitalizations in Germany. Since 2004, German hospitals receive compensation based on Diagnosis Related Groups (DRG). As per § 21 KHEntgG, it is mandated by law that German hospitals share data on all hospital discharges with the Hospital Remuneration Institute (InEK). After passing plausibility checks, anonymized data is forwarded to the Federal Statistical Office (FSO). Upon request, scientists can gain access to this dataset or subsets at regional research data centers.

### Case selection

Cases > 28 days and < 18 years of age hospitalized between 2016 and 2022 were analyzed. Cases were classified into non-surgical and surgical cases based on operation and procedure codes (OPS) that indicate surgery. Neonates were excluded because the planned reform does not affect neonatal care and because they represent a particularly complex group within the field of pediatrics.

### Determination of the treating department

The treating department was extracted from department codes. Cases with only pediatric department codes were classified as PD, cases with only adult department codes as AD, and cases that received pediatric and adult department codes within the same hospital stay as interdisciplinary (ID) cases. Department codes consist of four digits, of which five 2-digit combinations are specifically indicate pediatric departments: “10” = General pediatrics, “11” = Pediatric cardiology, “12” = Neonatology, “13” = Pediatric surgery, “30” – Pediatric and adolescent psychiatry. These 2-digit combinations can be leading (e.g., 10xx, 12xx) or tailing (e.g., xx10, xx12). All other department codes were considered as belonging to adult departments and explicitly coded as such in order to allow differentiation between PD, AD, and ID cases.

### PCCC score - medical complexity of underlying conditions

The second version of the Pediatric Complex Chronic Conditions (PCCC) system [12] was used to determine the complexity of the case deriving from chronic conditions. The PCCC was designed to identify and quantify chronic conditions in children that are likely to persist for at least one year. It consists of 10 categories representing organ systems plus one category each for conditions originating from the neonatal period and technology dependence. All categories are set to 0 by default - if one or more diagnoses are present, the corresponding category changes to 1. The total score is derived by building the sum of all categories with a possible maximum score of 12 according to the 10 + 2 categories. We used the previously described method with the same minor modifications due to lack of information on device prescription in the GHD [13].

### Modified PCCC score – accounting for incremental complexity with increasing number of underlying conditions

Because the original PCCC system employs linear summation of affected organ systems, the exponential increase of clinical complexity and treatment burden associated with multi-system involvement is insufficiently represented. Increasing complexity leads to more resource utilization including admissions, days, and charges [14], and the authors’ clinical experience shows that managing patients with multiple chronic (and acute) conditions is disproportionately more complex than managing patients with only one or two conditions. We therefore developed a mathematical framework to account for this incremental complexity by employing a logistic S-curve function on the PCCC sum score.

The modification employed the formula

$$\:{PCCC}_{mod}=\:\frac{M}{(1+{e}^{\left(-k\left(n-{x}_{0}\right)\right)})\:}$$

where.

M = 12 (maximum score).

n = original PCCC count (0–12 organ systems (10 organ systems + neonatology and technology dependence)).

x₀ = 3.5 (inflection point indicating the transition from simple to complex cases and reaching the half-maximum score).

k = 1.2 (steepness of the curve).

Key assumptions and goals that informed the parameters of the mathematical framework were:

- Clinical complexity follows an S-shaped curve with three phases:
  Low complexity (0–2 categories positive): Gradual increase, manageable cases.
  Escalating complexity (3–5 categories positive): Steepest complexity increase.
  High complexity plateau (6 + categories positive): all cases are maximally complex.
- Patients with no complex chronic conditions (PCCC = 0) should maintain a score of 0, preserving the heavy-tail distribution at baseline and the maximum score should be 12 as in the original PCCC.
- Based on clinical experience, the steepest increase of complexity occurs around 3–4 affected organ systems and therefore provides a clinically meaningful inflection point (x_0_) with the steepest increase of complexity, after which the complexity increases more slowly. The steepness of the S-curve should preserve the differentiation of scores for intermediate values avoiding overly rapid progression from very low scores to very high scores. Based on visual inspection of a family of curves for k = 1.0 (original score), 1.2, 1.5, 1.8, and 2.0, curves above 1.2 caused strong separation of score values (Supplementary Fig. 1a and 2). For that reason the inflectionpoint was set to x_0_ = 3.5.

Importantly, the modified score is based on expert assumptions and is not intended to replace validated scores but to explore nonlinear complexity effects.

### Primary and secondary outcomes

The unit of analysis was a case discharged from a German hospital between 2016 and 2022. The primary outcome was the proportion of non-surgical and surgical cases treated per department (PD versus AD). The secondary outcome was the normalized complexity ratio per 1000 cases between PDs and ADs for the original and the modified PCCC overall and stratified by surgery/non-surgery. To assure transparency and comprehensiveness, all primary and secondary outcomes were also calculated for ID cases.

### Missing data

There were no missing data on age and all cases had at least one diagnosis code and one department code. Missing surgical procedure codes could not be detected because there is no possibility to distinguish whether the surgery was not performed or not coded. However, surgical interventions are highly relevant for reimbursement, making missing codes unlikely. Likewise, missing codes on secondary diagnoses could not be detected but we relied on the assumption that relevant chronic conditions were coded, as higher complexity increases reimbursement for a case. The dataset does not include DRG cost weights or case-mix indices, and therefore acute severity or reimbursement-based case intensity could not be directly assessed.

### Statistical analysis

Frequencies were summarized as counts and percent, whereas continuous variables are presented as median and interquartile range (IQR) if skewed and mean ± standard deviation (SD) if distributed symmetrically and unimodal. Descriptive analyses were conducted overall and with the treating department, age, and surgery as grouping variables.

The system-level impact of modifying the PCCC was assessed by calculating the cumulative total scores and corresponding inflation ratios (by dividing the modified PCCC with the original PCCC) overall and for each department. To compare the relative chronic complexity of cases between departments descriptively, a normalized complexity score per 1000 admissions was calculated for the original and modified PCCC scores. To quantify the differences of chronic complexity of AD and ID cases, the normalized complexity ratio was calculated by dividing the normalized complexity of PD cases by that of AD and ID cases. The normalization and ratio building were performed for the original and modified PCCC scores.

### Software

SAS Version 9.4 (SAS Institute, Cary, USA) was used to analyze data at the regional research data center. Python 3.13 (Python Software Foundation, Beaverton, USA) in a Jupyter Lab environment (Version 4.3.4; Project Jupyter) [15] was used for final calculations and to produce figures.

### Ethics approval

No ethics approval was required according to local legislation because we used exclusively anonymized secondary health care data.

## Results

**Fig. 1:**
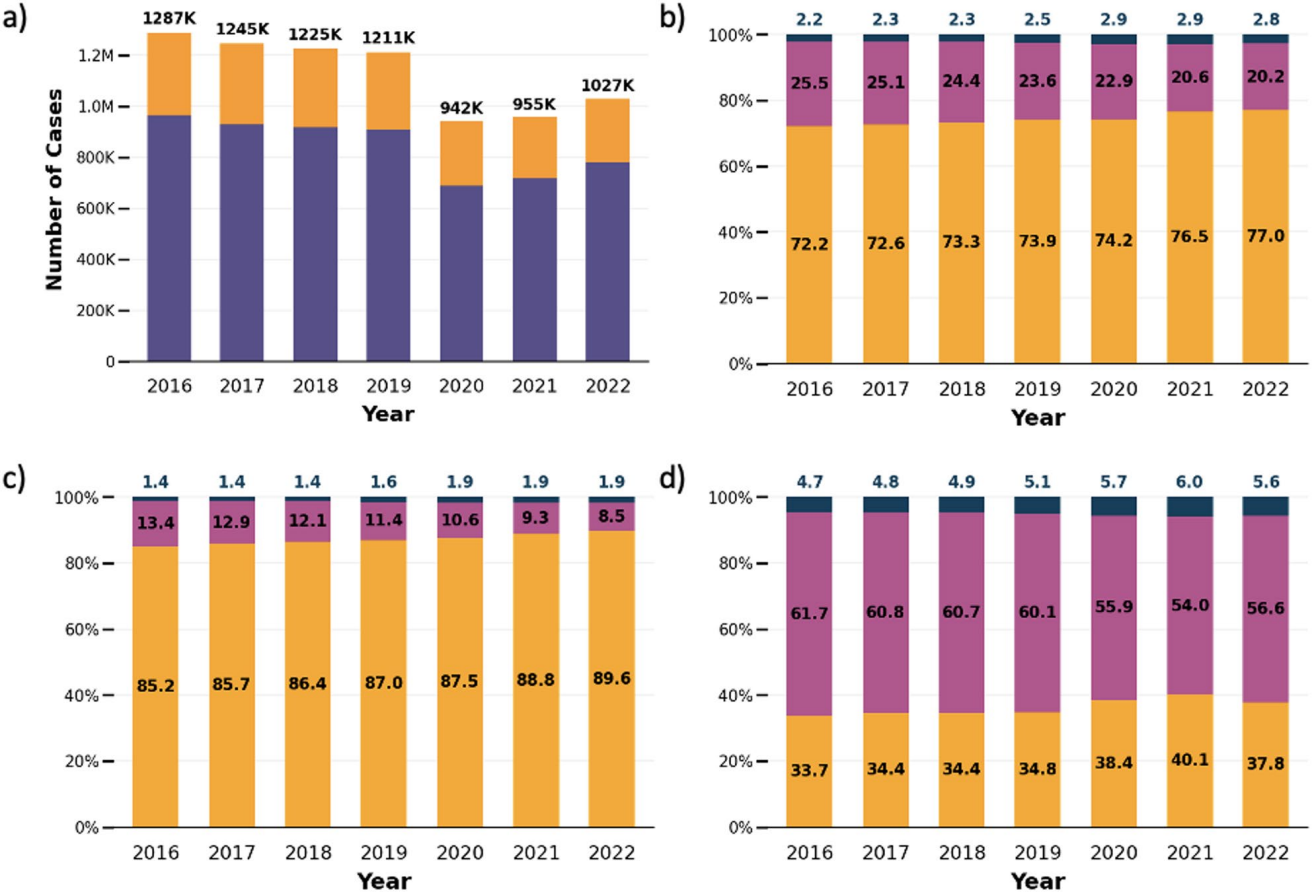
Pediatric hospital cases in Germany between 2016 and 2022 by year. **a **Total number of cases in pediatric departments (purple) and surgical departments (yellow). **b** Relative distribution of all cases between PD (yellow), AD (pink), and ID cases (blue). **c** Relative distribution of non-surgical cases between PD (yellow), AD (pink), and ID (blue). **d** Relative distribution of surgical cases between cases treated in PD (yellow), AD (pink), and ID (blue) PD = pediatric department, AD = adult department, ID = interdisciplinary cases

The original dataset contained 13,469,821 cases, of which 5,573,538 (41.4%) were neonatal and thus excluded. A total of 7,896,283 cases aged > 28 days were analyzed, of which 74.1% were treated in PDs, 23.4% in ADs, and 2.5% were interdisciplinary (Table 1). The total number of cases per year declined from 1,287,047 in 2016 to 1,027,934 in 2022, with a marked drop in 2020 (Fig. 1a). During the same period, AD cases declined from 25.5% in 2016 to 20.2% in 2022 overall (Fig. 1b) and also decreased when analyzing non-surgical and surgical cases separately (13.4 to 8.5% and 61.7 to 56.6%, respectively) (Fig. 1c and d).

**Table 1:** Case characteristics by treating department (pediatric hospital discharges in Germany, 2016–2022)

	Total	Pediatric	Adult	Interdisciplinary
Total, *n* (row %)	7,896,283 (100)	5,850,588 (74.1)	1,846,359 (23.4)	199,336 (2.5)
Non-surgical, *n (column %)*	5,896,533 (74.7)	5,131,535 (87.0)	669,429 (36.3)	95,569 (47.9)
Surgical, *n (column %)*	1,999,750 (25.3)	719,053 (12.3)	1,176,930 (63.7)	103,767 (52.1)
Gender				
Female, *n (column %)*	3,683,794 (46.7)	2,735,576 (46.8)	860,908 (46.6)	87,310 (43.8)
Male, *n (column %)*	4,212,240 (53.3)	3,114,830 (53.2)	985,391 (53.4)	112,019 (56.2)
Unknown, *n (column %)*	249 (0.003)	182 (0.003)	60 (0.003)	7 (0.003)
Intensive care unit admission, *n (column %)*	116,942 (1.5)	83,923 (1.4)	12,345 (0.7)	20,674 (10.4)
Mechanical ventilation	75,479 (0.96)	58,010 (0.99)	8,364 (0.45)	9,105 (4.6)
Duration of mechanical ventilation (days), *median (IQR)*	60 (20–155)	64 (24–158)	42 (8–142)	57 (17–152)
PCCC score, median (IQR)	0 (0–0)	0 (0–0)	0 (0–0)	0 (0–0)
PCCC = 0, *n (column %)*	6,229,237 (78.9)	4,443,538 (75.9)	1,639,556 (88.8)	151,143 (75,8)
PCCC = 1, *n (column %)*	1,118,578 (14.2)	945,237 (16.2)	147,419 (8.0)	25,922 (13,0)
PCCC ≥ 2, *n (column %)*	548,468 (6.9)	466,813 (7.8)	59,384 (3.2)	22,271 (11,2)
Outcomes				
In-hospital death, *n (column %)*	7,132	5,389 (0.09)	1,123 (0.06)	620 (0.31)
Non-surgical cases, *n (column %)*	4,715	3,829 (0.00)	647 (0.10)	239 (0.25)
Surgical cases, *n (column %)*	2,417	1,560 (0.22)	476 (0.04)	381 (0.37)
Length of stay all cases (days), *median (IQR)*	2 (1–3)	2 (1–4)	2 (1–3)	3 (1–6)
Length of stay non-surgical cases (days), *median (IQR)*	2 (1–4)	2 (1–3)	2 (1–3)	2 (1–3)
Length of stay surgical cases (days), *median (IQR)*	2 (1–3)	3 (1–5)	2 (1–4)	4 (2–8)

**Fig. 2:**
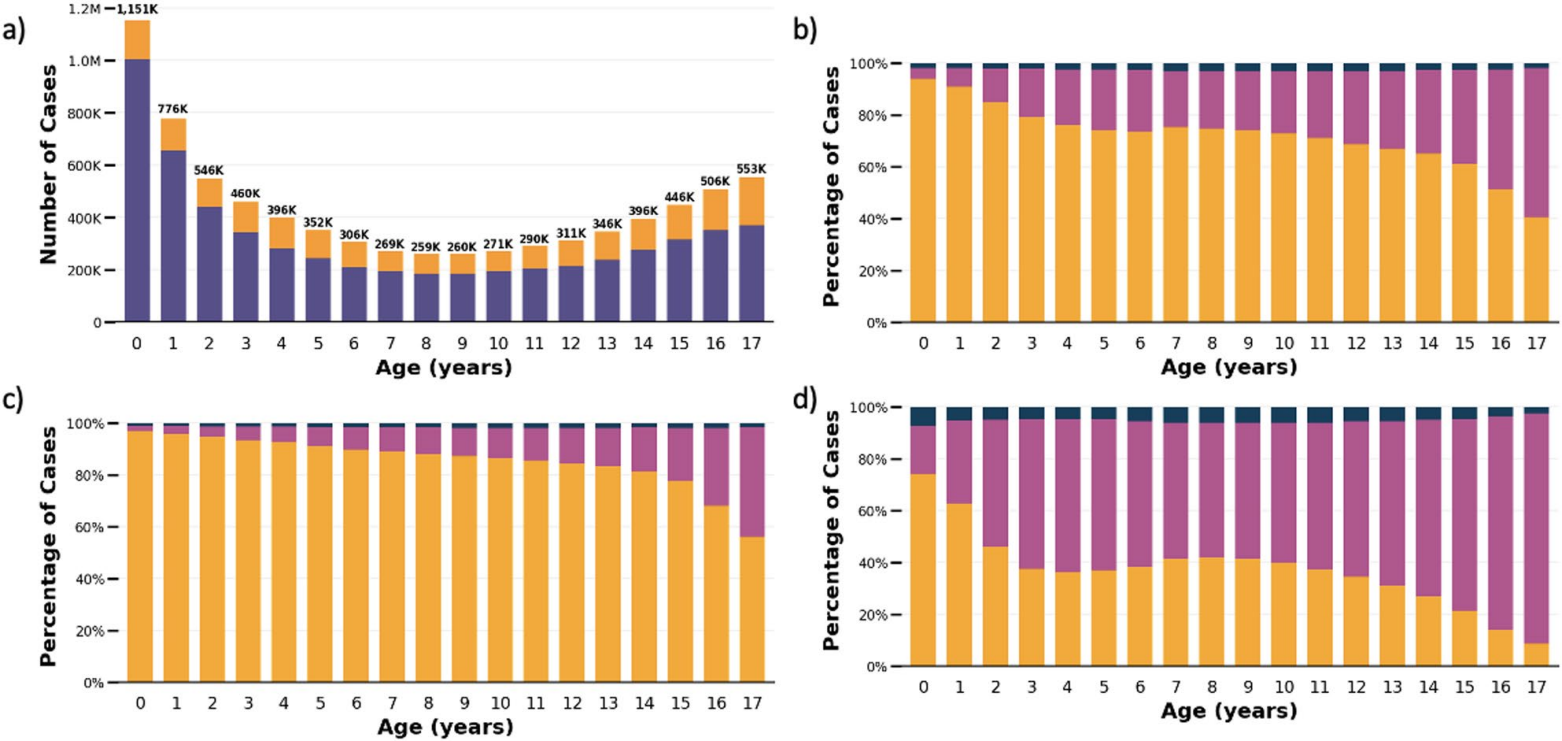
Pediatric hospital cases in Germany between 2016 and 2022 by admission age. **a** Total number of cases in pediatric departments (purple) and surgical departments (yellow). **b** Relative distribution of all cases between cases treated in PD (yellow), AD (pink), and ID (blue). **c** Relative distribution of non-surgical cases between cases treated in PD (yellow), AD (pink), and ID (blue). **d** Relative distribution of surgical cases between cases treated in PD (yellow), AD (pink), and ID (blue) PD = pediatric department, AD = adult department, ID = interdisciplinary cases

Of 5,896,533 non-surgical cases, 87.0% were treated in PDs, 11.4% in ADs, and 1.6% were ID cases. Out of 1,999,750 (25.3%) surgical cases, 58.9% were treated in ADs, while PD and ID cases accounted for 36.0% and 5.2%, respectively. The total number of admissions followed a U-shaped distribution with an increasing number and proportion of non-surgical and surgical cases treated in ADs at older ages (Fig. 2a-d).

Gender distribution did not differ between treating departments and hospital length of stay varied only slightly between departments and surgical/non-surgical cases (Table 1). Intensive care unit (ICU) admissions occurred in 1.4% of PD and 0.7% of AD cases. Case fatality was generally low with a total of 7,132 cases (0.09%) and similar between ADs and PDs. However striking differences from these findings were observed for the ID group with 10.4% ICU admission, 0.3% case fatality, and a tendency towards longer lengths of hospital stay (Table 1).

**Fig. 3:**
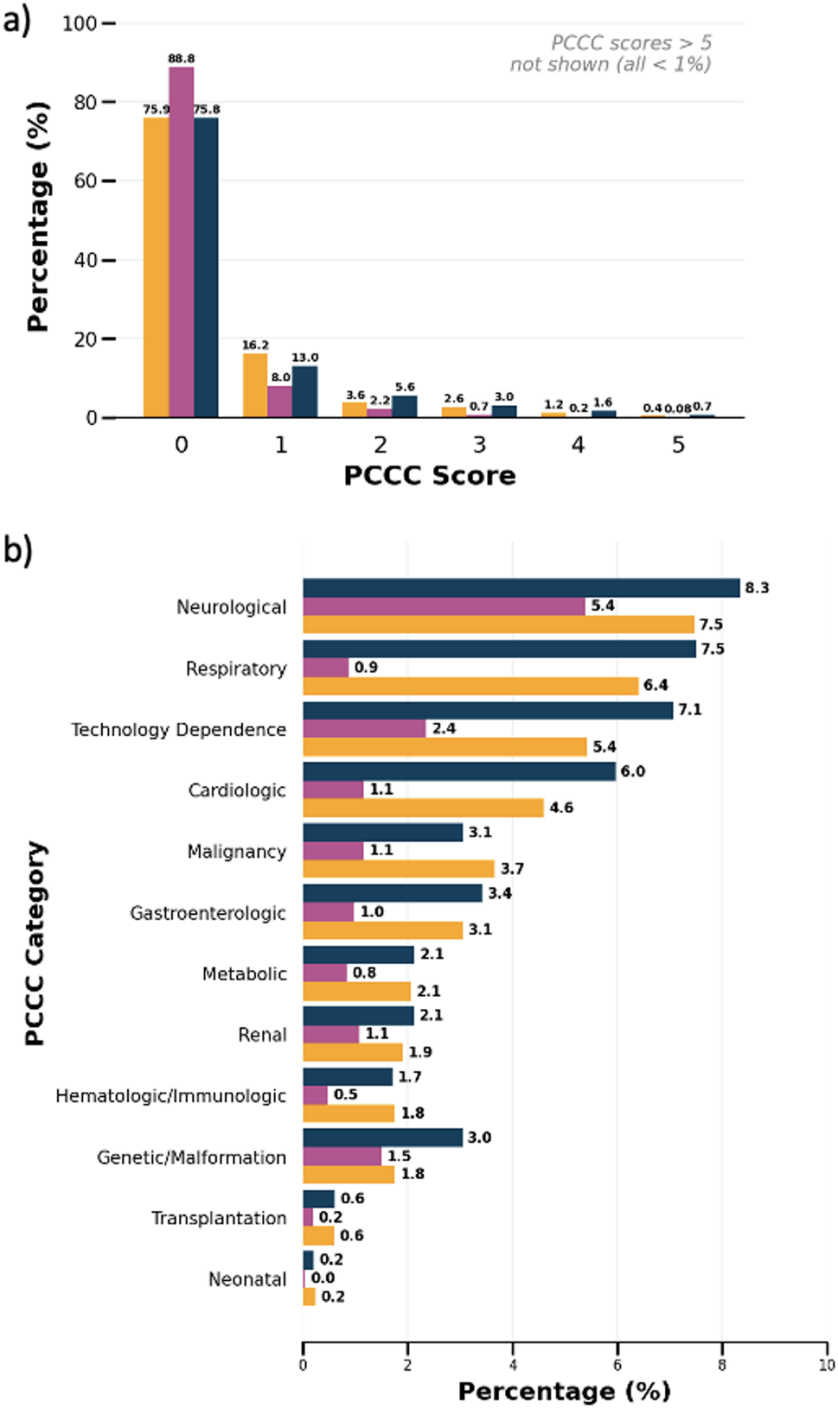
Pediatric complex chronic conditions (PCCC) score in pediatric hospital in cases in Germany between 2016 and 2022. **a** Distribution of PCCC scores of cases treated in treated in PD (yellow), AD (pink), and ID (blue) **b** Presence of PCCC categories of cases treated in PD (yellow), AD (pink), and ID (blue) PD = pediatric department, AD = adult department, ID = interdisciplinary cases

The case complexity analysis found that most treated cases had no or a very low burden of chronic conditions (Fig. 3a). The most frequently positive categories of the PCCC were neurological, respiratory, and technology dependence (Fig. 3b). The modification of the PCCC caused an inflation of the overall cumulative complexity by the factor 1.050 (Supplementary Table 1, Supplementary Fig. 1b). This inflation differed between departments (Supplementary Table 1, Supplementary Fig. 1c) with the highest observed inflation in ID cases (1.174), followed by PD cases (1.066). For ADs cases, the modification of the score caused a deflation of the cumulative complexity (0.892) (Supplementary Table 1).

The normalized complexity per 1000 cases differed between departments. ID cases had the highest normalized complexity using both PCCC versions, followed by PDs and ADs (Supplementary Table 1). To quantify the differences of complexity between departments, normalized complexity ratios were calculated comparing PD cases with AD and ID cases, respectively (Table 2). These ratios were above 1 comparing PDs with ADs, indicating higher complexity in PD cases. Subgroup analyses revealed higher ratios (= larger differences) for non-surgical compared to surgical cases, with higher complexity in PDs. Compared to ID cases, PD cases were less complex, holding true for the overall cohort and for the non-surgical/surgical subgroups (Table 2). All findings were more pronounced when using the modified PCCC compared to the original PCCC.

**Table 2:** Normalized complexity ratios between departments

	Original PCCC	Modified PCCC
Overall		
Pediatric vs. adult	2.43 (2.42–2.45)	2.90 (2.88–2.94)
Pediatric vs. interdisciplinary	0.86 (0.85–0.87)	0.78 (0.77–0.79)
Non-surgical		
Pediatric vs. adult	1.79 (1.77–1.81)	2.10 (2.07–2.13)
Pediatric vs. interdisciplinary	1.14 (1.12–1.16)	1.09 (1.07–1.12)
Surgical		
Pediatric vs. adult	3.66 (3.63–3.71)	4.98 (4.90–5.06)
Pediatric vs. interdisciplinary	0.86 (0.84–0.87)	0.85 (0.83–0.87)

Sensitivity analyses confirmed that reasonable variation of slope steepness for the modified PCCC did not change the direction of the relative complexity differences between departments.

## Discussion

This nationwide analysis of more than 7.8 million pediatric hospital cases in Germany between 2016 and 2022 found that most pediatric cases were treated in pediatric departments, reflecting baseline care patterns prior to the planning of the current hospital reform. More than 80% of non-surgical pediatric cases was treated in pediatric departments, while surgical cases were more frequently treated in adult departments. Case complexity, measured by the original and modified PCCC scores, was consistently higher in PDs compared to ADs, with interdisciplinary cases showing the highest overall complexity. These findings illustrate distinct patterns in care distribution and highlight the essential role of pediatric departments in managing pediatric cases with complex chronic conditions.

In addition to higher chronic complexity scores, pediatric departments also accounted for a greater proportion of ICU admissions and mechanical ventilation, indicating substantial resource intensity. Interdisciplinary cases showed the highest ICU utilization and mortality, underscoring their particularly high clinical and organizational burden. Length of stay differed only modestly between departments, suggesting that complexity differences are not solely reflected by duration of hospitalization.

Existing literature confirms that children—particularly those requiring surgical or trauma care—frequently receive treatment in adult departments: Several studies have shown that children with polytrauma or traumatic brain injury have higher mortality and poorer outcomes when treated in adult trauma centers [16–20]. A meta-analysis similarly reported increased odds of mortality, greater use of computed tomography, and more frequent operative management of solid organ injuries in pediatric trauma patients treated in adult settings [21]. While this evidence concerns trauma and surgical care, we found no studies evaluating outcomes of non-surgical pediatric inpatients treated in adult departments. Limited data, such as a study reporting higher satisfaction among adolescents treated on pediatric wards [22], suggest that the treatment setting may influence patient experience. The lack of literature on outcomes of non-surgical pediatric care in adult settings may indicate a research gap or that this care scenario is uncommon internationally.

Conversely, an increasing number of adults with chronic conditions originating in childhood continue to receive care in pediatric settings. These patients present to pediatric outpatient departments [23], emergency departments [24], general wards [25], and even pediatric intensive care units [26]. Their needs differ substantially from typical pediatric patients, involving higher comorbidity and treatment complexity. In response to these challenges, some institutions in the U.S. have implemented specialized adult inpatient services within pediatric hospitals, combining pediatric and adult expertise to close transitional care gaps [27]. This approach is supported by evidence showing adverse outcomes after transition to adult care: young people with life-limiting conditions present more frequently to emergency departments [28], and those with long-term conditions have increased emergency admissions following transition [29]. At the same time, structural debates have emerged in the U.S., as consolidation of hospital systems and declining pediatric inpatient capacity have led to closures of pediatric units and increased travel distances to pediatric services, raising concerns about access to specialized care [30, 31]. However, centralization is not equal to loss of specialty and, depending on the context, can drastically improve patient outcomes [32].

Against this backdrop, our study adds quantitative evidence to the ongoing debate surrounding Germany’s planned hospital reform and the proposed restructuring of pediatric specialty care. The argument that pediatric subspecialty services are dispensable because many children are already treated in adult departments is not supported by our results. Pediatric departments treat the majority of children, especially those with complex non-surgical conditions, and demonstrate higher case complexity overall. While part of this may be possibly attributed to different coding practices between departments, it likely reflects structural case-mix effects (e.g., post-trauma/post-operative monitoring in AD without chronic comorbidity) and therefore should be interpreted as differences in care distribution rather than causal effects of department type on outcomes. Importantly, lower chronic complexity does automatically not imply lower acute severity. However, ICU admission and mechanical ventilation were more frequent in pediatric departments than in adult departments, suggesting that higher chronic complexity was not offset by lower acute care intensity.

Importantly, the wide range of pediatric subspecialties—from cardiology and neurology to nephrology—differs fundamentally from structures in adult medicine. Yet, the reform consolidates this diversity into a single category of general pediatrics (besides neonatology and hemato-oncology), meaning that highly specialized pediatric services will no longer be distinctly designated or planned for. Our data indicate that complex pediatric cases are primarily managed in pediatric departments, reflecting established subspecialized care structures. Restricting the care group framework to general pediatrics may therefore insufficiently represent existing treatment patterns, particularly for children with multi-system chronic conditions or rare diseases requiring coordinated specialized care. These findings suggest that, beyond readiness financing, the structural recognition of pediatric subspecialty services warrants careful consideration within the reform process, especially in light of increasing chronicity and the growing number of young adults with pediatric-onset conditions. While overflow to adult care may be appropriate for less complex cases or during capacity constraints, pediatric departments concentrate a disproportionate share of higher-complexity patients, implying greater demands for specialized personnel, coordination, and infrastructure. Restructuring efforts should therefore account for this case-mix distribution to ensure alignment between resource allocation and clinical burden. Structured models that safeguard age- and complexity-appropriate care remain essential.

### Limitations and future research

Limitations of this study include the need to adapt the PCCC score due to missing device-related codes in the GHD, and the impossibility to assess acute case severity due to lack of information on DRG weights in the GHD. Further, the identification of departments relied on administrative coding, which does not reflect dedicated teams operating within a superimposed department, e.g. pediatric teams in adult departments or vice versa. The PCCC system captures only chronic conditions and may underestimate the burden in cases with multiple conditions from a single organ system and/or acute disease-related complexity but cannot be interchangeably used with acute severity. Due to lack of information in the German modification of the ICD-10 system, we could not assess whether a chronic condition was pre-existing or acquired during the hospital stay. Geographical difference were not analyzed and the socioeconomic status was not available in the dataset. Surgical cases were defined as cases undergoing surgery, but some cases may have been managed in surgical departments without receiving operative interventions, e.g., monitoring after traumatic brain injury. Interdisciplinary cases represent a small but clinically relevant group with the highest PCCC scores, highest proportion of intensive care unit admissions and highest case fatality rate. Possibly, these cases represent a subgroup of patients with particularly high medical and organizational complexity, necessitating cross-departmental expertise, e.g., children with multi-system chronic disease and superimposed acute severity. These findings emphasize that structural interfaces between departments require careful consideration in the reform process.

Future research should include dedicated outcome-oriented analyses comparing pediatric and adult care settings, particularly for high-risk patient groups.

## Conclusion

Overall, our findings underscore the indispensable role of pediatric departments in managing complex care and support the need for structured, patient-centered reforms that preserve specialized pediatric expertise.

## Supplementary Information

Supplementary Material 1.

Supplementary Material 2.

## Data Availability

The original dataset remains at the Federal Statistical Office and can be accessed by qualified researchers at designated research data centers after filing a request and signing a confidentiality agreement. The data generated for this study and exported from the Federal Statistical Office will be made available upon reasonable request.

## Abbreviatons

AD: Adult department
DRG: Diagnosis Related Groups
FSO: Federal Statistical Office
GHD: German hospital dataset
ICD-10: International Classification of Diseases, 10th Revision
ICU: Intensive care unit
ID: Interdisciplinary
InEK: Institute for the Hospital Remuneration System (Institut für das Entgeltsystem im Krankenhaus)
IQR: Interquartile range
OPS: Operation and Procedure Codes (Operationen- und Prozedurenschlüssel)
PCCC: Pediatric Complex Chronic Conditions
PD: Pediatric department
SD: Standard deviation
