# Supplementary material for: The frequency and complexity of pediatric hospitalizations to pediatric and adult departments in Germany
Source: BMC Pediatr. 2026 Apr 8;26:318. doi: 10.1186/s12887-026-06816-4 (PMC13081376; doi:10.1186/s12887-026-06816-4)
Supplement: Supplementary file 1 — Supplementary Material 1. [file 12887_2026_6816_MOESM1_ESM.docx]

**Supplementary Table 1:** Cumulative complexity and inflation factors of the modified compared to the original PCCC score

| **Group** | **Number of cases** | **Cumulative complexity** | | **Inflation factor** | **Normalized complexity** /1000 cases (95 % CI) | |
| --- | --- | --- | --- | --- | --- | --- |
|  |  | **Original PCCC** | **Modified PCCC** |  | **Original PCCC** | **Modified PCCC** |
| Overall | 7,896,283 | 2,659,553 | 2,792,398 | 1.050 |  |  |
| Pediatric | 5,850,588 | 2,274,688 | 2,423,763 | 1.066 | 388.8 (388.1 – 389.5) | 414.3 (413.2 – 415.4) |
| Adult | 1,846,359 | 294,902 | 263,023 | 0.892 | 159.7 (159.0 – 160.5) | 142.5 (141.5 – 143.4) |
| Interdisciplinary | 199,336 | 89,963 | 105,612 | 1.174 | 451.3 (446.9 – 455.7) | 529.8 (522.7 – 536.9) |

**Supplementary Table 2:** Cumulative complexity and inflation factors of the modified compared to the original PCCC score depending on intensive care unit admission

| **ICU admission** | **Group** | **Number of cases** | **Cumulative complexity** | | **Inflation factor** | **Normalized complexity**  /1000 cases (95 % CI) | |
| --- | --- | --- | --- | --- | --- | --- | --- |
|  |  |  | **Original PCCC** | **Modified PCCC** |  | **Original PCCC** | **Modified PCCC** |
| No ICU admission | Overall | 7,779,341 | 2,444,859 | 2,752,856 |  |  |  |
|  | Pediatric | 5,766,665 | 2,115,294 | 2,198,148 | 1.039 | 366.8 (366.1 – 367.5) | 381.2 (380.2 – 382.2) |
|  | Adult | 1,834,014 | 274,775 | 237,834 | 0.866 | 149.8 (149.1 – 150.5) | 129.7 (128.8 – 130.6) |
|  | Interdisciplinary | 178,662 | 54,790 | 59,221 | 1.081 | 306.7 (303.0 – 310.4) | 331.5 (325.8 – 337.1) |
| ICU admission | Overall | 116,942 | 2,146,694 | 324,484 |  |  |  |
|  | Pediatric | 83,923 | 159,394 | 225,616 | 1.416 | 1,899.3 (1888.2 – 1910.4) | 2,688.4 (2665.5 – 2711.2) |
|  | Adult | 12,345 | 20,127 | 25,189 | 1.252 | 1,630.4 (1604.8 – 1656.0) | 2,040.4 (1990.5 – 2090.3) |
|  | Interdisciplinary | 20,674 | 35,173 | 46,391 | 1.319 | 1,701.3 (1680.5 – 1722.1) | 2,243.9 (2203.1 – 2284.7) |
