# Supplementary material for: The frequency and complexity of pediatric hospitalizations to pediatric and adult departments in Germany
Source: BMC Pediatr. 2026 Apr 8;26:318. doi: 10.1186/s12887-026-06816-4 (PMC13081376; doi:10.1186/s12887-026-06816-4)
Supplement: Supplementary file 2 — Supplementary Material 2. [file 12887_2026_6816_MOESM2_ESM.docx]

**
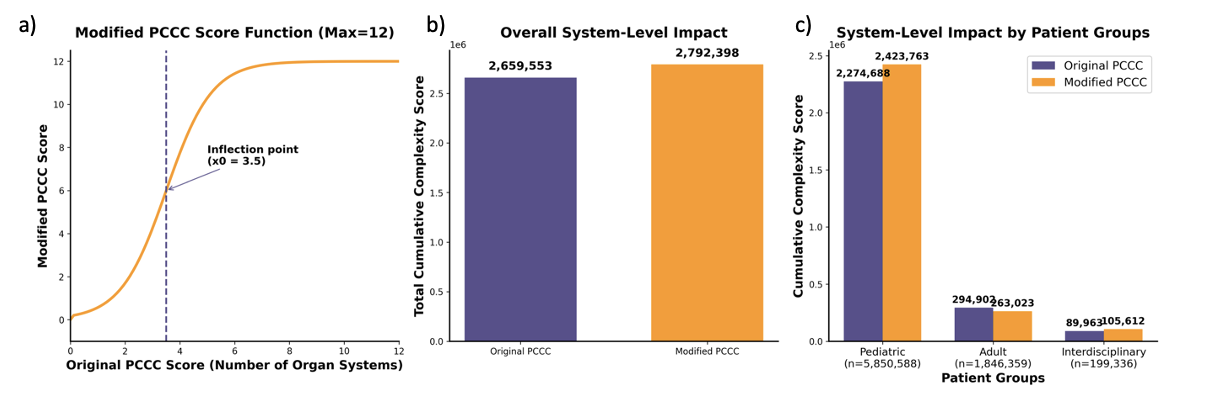
**

**Supplementary Figure 1:** Modification of the pediatric complex chronic conditions (PCCC) score

a) Modifcation of the PCCC score using the S-shaped curve to correct for increasing complexity in multiple conditions

b) System level impact of the PCCC score modification on the cumulative complexity of all cases

c) System level impact of the PCCC score modification on the cumulative complexity by department


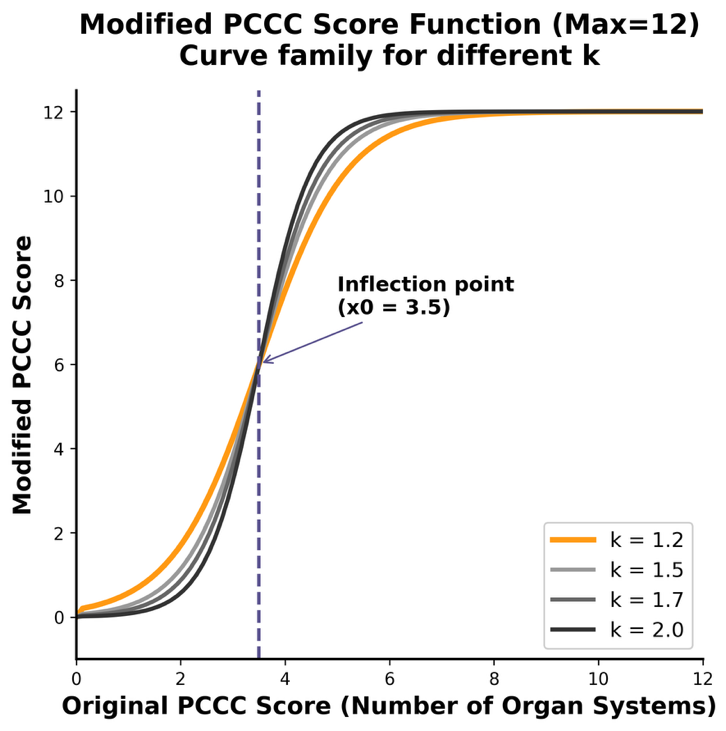


**Supplementary Figure 2:** Family of curves with differing k-values for the modification of the pediatric complex chronic conditions (PCCC) score
